# Functional characterization of TMEM86A and TMEM86B mutants by a novel lysoplasmalogenase assay

**DOI:** 10.1016/j.jlr.2025.100766

**Published:** 2025-02-28

**Authors:** Denise Kummer, Ilaria Dorigatti, Theresia Dunzendorfer-Matt, Georg Golderer, Ernst R. Werner, Katrin Watschinger

**Affiliations:** Institute of Molecular Biochemistry, Biocenter, Medical University of Innsbruck, Innsbruck, Austria

**Keywords:** enzymology, glycerophospholipids, lipids, lipid degradation, lysoplasmalogens, phospholipids/metabolism, plasmalogens

## Abstract

Plasmalogens are an abundant class of glycero-phospholipids with a characteristic 1-*O*-alk-1′-enyl double bond. While their synthesis has been extensively investigated, their degradation remains understudied. Plasmalogen deficiencies are associated with severe disorders in humans and interfering with their degradation would be a treatment option, but it remains out of reach due to limited knowledge. The plasmalogen double bond is degraded either directly by a plasmalogenase or by conversion to the 2′ lyso forms by phospholipase and subsequent cleavage by lysoplasmalogenase (E.C. 3.3.2.2). Two lysoplasmalogenases are known so far, TMEM86A and TMEM86B. While TMEM86B has been expressed in bacteria, purified, and shown to encode lysoplasmalogenase activity by a coupled optical assay, the closely related protein TMEM86A has not yet been purified, but its activity was shown indirectly by a lipidomics approach. Here, we present a novel assay for lysoplasmalogenase activity based on incubation with lysoplasmenylethanolamine or lysoplasmenylcholine, derivatization of the aldehyde product with dansylhydrazine, and hydrazone quantification by reversed-phase HPLC with fluorescence detection. The method was sensitive enough to robustly detect lysoplasmalogenase activity in human embryonic kidney cells following transient expression of TMEM86A or TMEM86B and also suitable for the determination of lysoplasmalogenase activity in mouse tissues where highest activities were found in liver and duodenum. We introduced point mutations at positions proposed to be catalytically relevant and provided experimental evidence that all but one affected lysoplasmalogenase activity. Our novel assay allows direct and fast measurement of lysoplasmalogenase activity, thereby providing a tool to advance research in the field of plasmalogen degradation.

Plasmalogens are an abundant class of glycero-phospholipids characterized by a 1-*O*-alk-*1′*-enyl double bond, which gives them special chemical and biochemical properties (1). They make up about 20% of the phospholipid pool in mammals and have been implicated in various human disorders including Zellweger syndrome, Rhizomelic Chondrodysplasia Punctata, but also neurodegenerative disorders such as Alzheimer's disease (1, 2). While their biosynthetic routes in peroxisomes and endoplasmic reticulum have been extensively investigated, their catabolism is still more enigmatic (1). Two distinct pathways have been discussed in literature (for review see (1)): either they are directly catabolized by cytochrome c, which has been shown to have plasmalogenase activity (3), or they are first converted to their 2′ lyso forms by phospholipase A2 and subsequently hydrolytically cleaved by the specialized enzyme lysoplasmalogenase (E.C. 3.3.2.2) at their characteristic 1-*O*-alk-*1′*-enyl double bond to yield the corresponding aldehyde product. In a work published in 2011, lysoplasmalogenase was purified from rat liver microsomes, and its enzymatic properties were determined. Partial sequences of the purified rat protein were obtained and it was assigned to Tmem86b, a sequence with several predicted hydrophobic transmembrane stretches belonging to the family of YhhN proteins (PFAM PF07947) (4). Sequence analysis also revealed a closely related protein of unknown function, TMEM86A, which in contrast to TMEM86B has not been purified so far. Based on sequence homology (4) and from a lipidomic analysis of tissues with altered TMEM86A expression, it was concluded in 2022 that this protein is also a lysoplasmalogenase (5). TMEM86A was identified in genome-wide screens to be involved in resistance to severe acute respiratory syndrome coronavirus 2 infection (6), as well as in plasmalogen homeostasis and protein-kinase A–dependent energy metabolism (5). In bone marrow–derived murine macrophages, Tmem86a was found to be a sterol-regulated lysoplasmalogenase that contributes to sterol-dependent membrane remodeling (7).

Further investigations on the enzymatic properties of the *Legionella pneumophila* YhhN protein revealed that it behaved very similarly to the mammalian protein regarding kinetic parameters, substrate specificity and pH optimum (8). The same study showed that bacterial YhhN had eight predicted transmembrane helices and eight putative active site residues, which were highly conserved among YhhN proteins including human TMEM86A and TMEM86B and were distributed across these predicted transmembrane regions. Two of these residues, aspartates 82 and 190, were mutated to alanine in TMEM86A, which led to a decrease of their activity quantified by LC-MS (5).

For a straightforward enzymatic characterization, an important prerequisite is a sensitive and reliable assay. Wu *et al.* used a coupled optical assay employing yeast alcohol dehydrogenase to characterize the purified TMEM86B protein (4). In this assay, the aldehyde product of the lysoplasmalogenase reaction was enzymatically reduced to an alcohol by this dehydrogenase, and stoichiometric oxidation of NADH was detected by UV absorption. Van Wouw *et al.* in 2023 were able to demonstrate lysoplasmalogenase activity close to its detection limit in TMEM86A-transfected HEK293T cells by an adaptation of this optical assay (7). An older assay relied on a radioactively labeled lysoplasmalogen and separation of radioactive products by thin layer chromatography (9).

Here, we present a novel sensitive assay for lysoplasmalogenase based on the detection of the aldehyde product in enzymatic incubation mixtures after derivatization to the highly fluorescent dansylhydrazone. Lipids of the reaction mixture were separated by reversed-phase HPLC, and the hydrazone was quantified by fluorescence detection. This allowed the quantification of lysoplasmalogenase activity in mouse tissues and transfected HEK293T cells. An advantage of this HPLC-based assay is the ability to rapidly measure and monitor activities, which we have also used to analyze the previously proposed enzymatically important amino acids (8). Our assay provides a fast and reliable tool to gain insights into the degradation of lysoplasmalogens, thereby advancing the field of plasmalogen catabolism. This is urgently needed as deficiencies in these lipids, which occur not only in congenital peroxisomal disorders in small infants but also in neurodegenerative syndromes such as Alzheimer's or Parkinson's disease in the elderly, could be ameliorated by interfering with plasmalogen degradation, opening up new treatment strategies.

## Materials and methods

### Reagents and cell lines

The lysoplasmalogenase substrates 1-*O*-1′-(Z)-octadecenyl-2-hydroxy-*sn*-glycero-3-phosphoethanolamine (LPE[P], 852471) and 1-*O*-1′-(Z)-octadecenyl-2-hydroxy-*sn*-glycero-3-phosphocholine (LPC[P], 852465) were obtained from Avanti Polar Lipids (Sigma, Vienna, Austria). All other chemicals were from Sigma (Vienna, Austria), Roth (Karlsruhe, Germany), or Serva (Heidelberg, Germany). Expression plasmids for human TMEM86A and TMEM86B in pCMV-Sport6 were from the Mammalian Genome Collection (Horizon, Cambridge). Cell lines were from ATCC (Manassas, VA).

### Cell culture, preparation and transfection of TMEM86A and TMEM86B mutants, and harvest of mouse tissues

HEK293T cells were grown in DMEM with high glucose (D6429, Sigma) containing 10% heat-inactivated fetal bovine serum (Gibco, Thermo Fisher Scientific, Vienna, Austria, A526701) at 37°C in a humidified 5% (v/v) CO_2_ atmosphere without addition of penicillin or streptomycin. For transfection, cells were seeded at a density of 5 × 10^5^ in 6-well plates and transfected on the next day with endotoxin-free plasmid DNA obtained with the EndoFree® Plasmid Maxi Kit (Qiagen, Hilden, Germany). For optimization of the activity assay, we diluted 4 μg human *TMEM86A* or *TMEM86B* plasmid DNA in the expression vector pCMV-Sport6 or pEGFP-N1 (Clontech, Mountain View, CA) as a control in 400 μl Optimem Reduced Serum Medium (Gibco) and 6 μl Turbofect (Thermo Fisher Scientific), vortexed for 10 s, and incubated for 20 min at room temperature, before adding it dropwise to the wells containing 2 ml culture medium. For analysis of point mutations, we introduced the desired sequence changes in wildtype human *TMEM86A* or *TMEM86B* in the CMV-promotor–driven expression vector pEXPR-IBA103 (IBA, Göttingen, Germany), harboring a C-terminal 6x myc-tag using the QuikChange*®* II site-directed mutagenesis kit (Agilent, Waldbronn, Germany) and the primers listed in Supplemental Table S1. The correctness of the mutated open reading frames was verified by sequencing (Microsynth, Balgach, Switzerland). For transfection, we employed the same procedure using 4 μg human *TMEM86A* or *TMEM86B* wildtype or mutants in pEXPR-IBA103 (pEGFP-N1 was again used as mock control). At 48 h post transfection, cells were harvested for activity assays or Western blotting by washing them with PBS, immediately snap-freezing the pellets in liquid nitrogen and storing them at −80°C until analyzed.

For lysoplasmalogenase activity tissue distribution, we used 12-week-old C57BL6/N wildtype mice (3 females and 3 males) from the animal facility of the Medical University of Innsbruck, which is approved by the Austrian Federal Ministry of Education, Science and Research (BMBWF-66.011/0017-II/3b/2014). Animals were euthanized by cervical dislocation, tissues were excised, immediately snap-frozen in liquid nitrogen and stored at −80°C until analysis.

### Homogenization of HEK293T cells and mouse tissues and membrane preparation of mouse tissues for lysoplasmalogenase activity assays

For cell lysis, 100–150 mg glass beads and 150 μl of glycylglycine (Gly/Gly) sucrose buffer (250 mM sucrose in 70 mM Gly/Gly, pH 7.2) were added to the stored pellet, vortexed and shaken in a mixer-mill MM 400 (Retsch, Haan, Germany) at 20 Hz, 4 times 30 s in a precooled block. Tissues in contrast were homogenized in 200–800 μl (depending on the size of the tissue) Gly/Gly sucrose buffer (250 mM sucrose in 70 mM Gly/Gly, pH 7.2) using an Ultra Turrax (IKA, Staufen, Germany). For the preparation of membranes from murine liver, subcutaneous white adipose tissue (sWAT) and visceral white adipose tissue (vWAT), tissues were homogenized in homogenization buffer (10 mM Tris–HCl pH 8.0 containing 1% protease inhibitor mix (Thermo Fisher Scientific) and 1 mM PMSF) using an Ultra Turrax. After 15 min incubation on ice, cells were disrupted by pushing the homogenate four times through a 0.4 × 20 mm needle. To remove cells and debris, homogenates were first centrifuged at 660 *g* for 20 min at 4°C. The resulting supernatant was then ultracentrifuged at 300,000 *g* for 20 min at 4°C to pellet the membranes, which were subsequently resuspended in 100 μl of homogenization buffer.

### Lysoplasmalogenase enzyme assay, lipid extraction, and derivatization

Homogenized cell and tissue samples were placed on ice. Protein concentration was determined by Bradford assay reagent (Bio-Rad, Vienna, Austria) using BSA as standard. All samples were diluted to 1 mg/ml (except for membrane preparation where a final concentration of 0.15–0.5 mg/ml was used) in 70 mM Gly/Gly pH 7.2 containing 0.5 mg/ml BSA. The assay mixture contained, in a total volume of 100 μl (final concentrations): 100 μM substrate (lysoplasmenylcholine C18-LPC[P] or lysoplasmenylethanolamine C18-LPE[P]), 1 mg/ml protein homogenate, 70 mM Gly/Gly pH 7.2, and 0.5 mg/ml BSA. The reaction was started by adding the diluted protein and stopped after 30 min at 37°C with 500 μl chloroform/methanol (2:1, v/v).

To extract the lipids, the stopped assay mix was vigorously shaken for 1 min, the organic phase was then transferred to a fresh low bind reaction tube (Eppendorf, Vienna, Austria) and the remaining aqueous phase was extracted with another 500 μl chloroform/methanol (2:1, v/v). After pooling of the so-obtained organic phases and centrifuging them for 2 min at 4°C and 17950 *g*, they were transferred to brown glass vials and the organic solvent was allowed to evaporate either overnight in a hood or under a stream of nitrogen. Thereafter, derivatization of the contained aldehydes was achieved after reconstitution of the lipids in 100 μl acetonitrile/ethanol (1:1, v/v). The solution was shaken at 37°C for 10 min and 10 μl of this lipid extract were incubated with 40 μl 1.7 mM dansylhydrazine (Sigma, 635928) in acetonitrile/2 M acetic acid (9.3/0.7, v/v) on ice in the dark for 15 min. This incubation was terminated by centrifugation at 20800 *g* at 4°C for 5 min.

For the optimization of lysoplasmalogenase assay conditions, some incubations were carried out for 0–60 min, the substrate concentrations were varied from 0 to 200 μM, the protein content from 0 to 3 mg/ml and the following buffers were tested: 70 mM Gly/Gly (pH 7.2, 7.6, and 8.0) and 70 mM potassium phosphate (pH 6.4, 6.8, and 7.2). In initial experiments, acyl-CoA–independent transacylase inhibitor SKF98625 (10, 11) was added to the assay mixtures containing liver homogenates in final concentrations ranging from 10 to 100 nM. To check for the impact of lipids in the extract interfering with aldehyde conversion to the hydrazone, 0, 0.3, 1, and 3 μM octadecanal were derivatized in presence or absence of a lipid extract prepared from HEK293T cells and analyzed by HPLC, as outlined below.

### HPLC analysis of reaction products

For HPLC analysis, samples were transferred to HPLC vials and placed in an Agilent 1200 HPLC system (Agilent Technologies, Vienna, Austria) equipped with a thermostatted autosampler (T = 10°C), fluorescence detector, and a column thermostat set to 25°C. At a flow rate of 1 ml/min and an injection volume of 10 μl, a Zorbax Eclipse XDB-C8 4.6 × 50 mm, 3.5 μm particle size column (Agilent Technologies) was eluted with 10 mM potassium phosphate buffer (pH 6.0) containing 79% (v/v) methanol for 3 min, followed by a linear gradient elution step to 100% methanol over 7 min. One hundred percent methanol was held for 5 min, and the column was then equilibrated to the starting condition for 2 min. Dansylhydrazones were detected by fluorescence (excitation 340 nm and emission 525 nm). As an external standard for the dansylhydrazone with 18 carbon atoms, we incubated in initial experiments 10 μl of 20 μM freshly dissolved plasmalogen 1-(1Z-octadecenyl)-2-oleoyl-*sn*-glycero-3-phosphoethanolamine (Sigma, 852758P) with 40 μl of 1.7 mM dansylhydrazine in acetonitrile/2 M HCl (9.3/0.7, v/v) on ice in the dark for 15 min to quantitatively release octadecanal and derivatize it to the corresponding hydrazone. Later, upon commercial availability, we used octadecanal (Merck, Vienna, Austria) for derivatization. Representative chromatograms for peak verification are shown in supplemental Fig. S1.

### Detection of myc-tagged protein levels by Western blotting

We quantified the amount of recombinant myc-tagged protein formed in order to be able to correct the measured lysoplasmalogenase activities for the potential impact of the point mutations on protein expression levels. Thus, 48 h after transfection, cells from wells transfected in parallel were harvested as explained above, suspended in 30–100 μl (depending on the size of the pellet) of 0.1 M Tris–HCl buffer, pH 7.6 containing 0.25 M sucrose, and the protein content was determined by the Bradford assay. 25–40 μg of protein was separated on Novex™ Tris-Glycine Mini Protein Gels, 4–20%, 1.0 mm, WedgeWell™ format (Thermo Fisher Scientific), blotted to PVDF membranes (Thermo Fisher Scientific), incubated with rabbit polyclonal anti-myc antibody (ab9106, dilution 1:4,000, Abcam, Cambridge, ab9106), washed, and incubated with a goat-anti-rabbit Cy5-labeled antibody (dilution 1:1,250, Cytiva PA45011, Fisher Scientific). Beta-actin was used as a loading control by staining with mouse anti-actin antibody (dilution 1:2,000, Sigma, MAB1501R) and goat anti-mouse Cy3 (dilution 1:1,250, Cytiva PA43009, Thermo Fisher Scientific). Blots were scanned using a red laser (633 nm excitation and 670 nm emission; BP30) for Cy5 and a green laser (532 nm excitation and 580 nm emission; BP30) for Cy3 with a Typhoon 9410 (GE Healthcare, Vienna, Austria). Blot signals were quantified by ImageQuant TL software (GE Healthcare), and the ratio between myc to actin signal was calculated and used to normalize the activity values obtained in the lysoplasmalogenase assay.

### Statistics

Results are presented as means ± SEM and analyzed with GraphPad Prism 10.2.3 by two-tailed unpaired *t* test or two-way ANOVA followed by Tukey posthoc test.

## Results

### A novel assay to monitor lysoplasmalogenase activities in cells and tissues

**Fig. 1:**
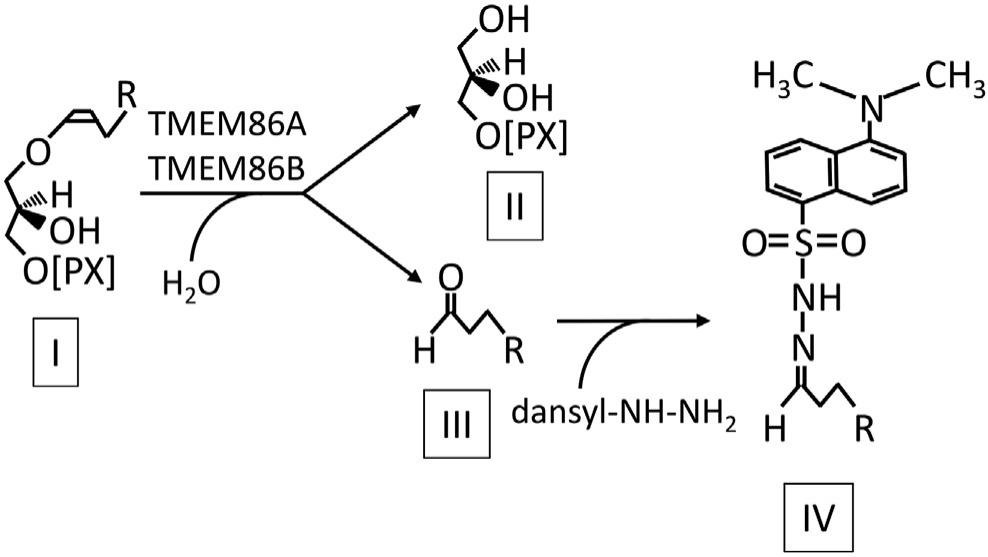
Reaction scheme of the lysoplasmalogenase assay. Lysoplasmalogen substrates (I) are cleaved by TMEM86A and TMEM86B in a hydrolytic cleavage, yielding a glycerol derivative (II) and a free aldehyde (III). To quantify the amount of cleaved plasmalogens, the so-formed aldehydes are converted to fluorescent hydrazones (IV) with dansylhydrazine (dansyl-NH-NH_2_). PX, phosphoethanolamine or phosphocholine; R, typical naturally occurring linear lipid chain.

We established a sensitive assay for lysoplasmalogenase activity. For this, we used two different commercially available lysoplasmalogen substrates (for details see section *reagents and cell lines* in Materials and Methods) and derivatized the aldehyde resulting from the lysoplasmalogenase reaction by incubation with dansylhydrazine, which converts the aldehyde to the corresponding fluorescent hydrazone (Fig. 1).

**Fig. 2:**
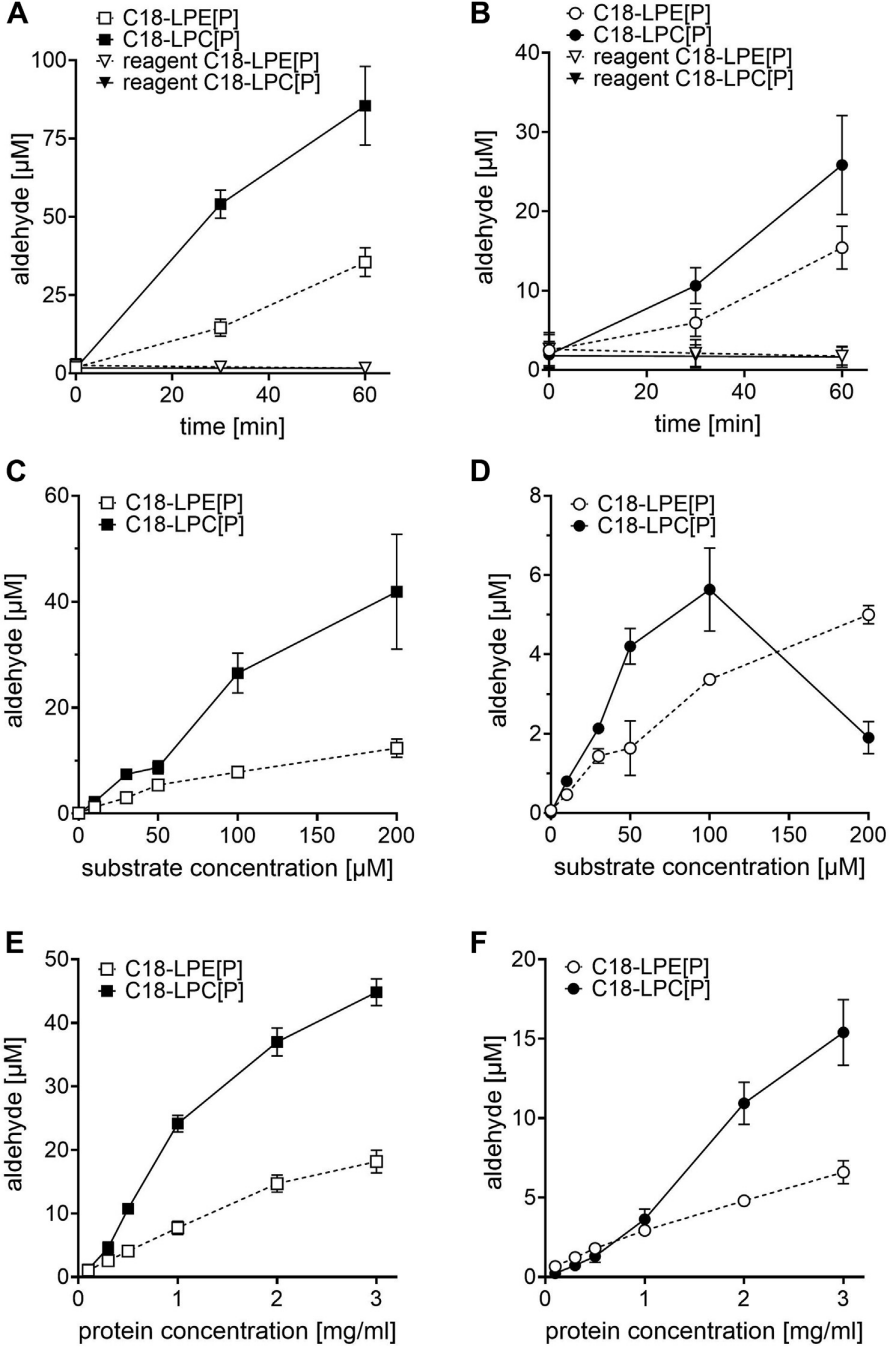
Hydrolytic cleavage of lysoplasmalogens by HEK293T cells after transfection with either human TMEM86A (A, C, and E) or human TMEM86B (B, D, and F) and derivatization of the formed aldehyde. A and B: Time dependence of lysoplasmalogen cleavage by human TMEM86A (A, squares) and TMEM86B (B, circles) using 100 μM of the substrates and 1 mg/ml protein homogenate. Open symbols and dashed lines represent the C18-LPE[P] substrate, filled symbols and solid lines represent the C18-LPC[P] substrate. Triangles represent the respective control experiments without any protein added. Lysoplasmalogens were cleaved by lysoplasmalogenase activities, lipids extracted, and aldehydes derivatized with dansylhydrazine to the respective fluorescent hydrazone. The amount of aldehyde was calculated from the area of the peak at the correct retention time. The mean ± SEM for three independent experiments is shown. C and D: Substrate concentration dependence of aldehyde formation by TMEM86A (C, squares) and TMEM86B (D, circles) using the C18-LPE[P] substrate (open symbols, dashed lines) or the C18-LPC[P] substrate (filled symbols, solid lines). The concentration of the used protein homogenate was 1 mg/ml and the assay was stopped after 30 min. The mean ± SEM for three independent experiments is shown. E and F: Dependence of the amount of aldehyde formed by human TMEM86A (E, squares) and TMEM86B (F, circles) on the amount of cellular protein homogenate using 100 μM C18-LPE[P] substrate (open symbols and dashed lines) or 100 μM C18-LPC[P] substrate (filled symbols and solid lines) and stopping the assay after 30 min. The mean ± SEM for three independent experiments is shown. LPC[P], lysoplasmenylcholine; LPE[P], lysoplasmenylethanolamine.

The reaction products were then separated by reversed-phase HPLC and the hydrazone amounts were detected using a fluorescence detector at an excitation of 340 nm and an emission of 525 nm. To establish standard assay conditions, we heterologously transfected both human TMEM86A and human TMEM86B, respectively, into HEK293T cells and determined the dependence of their enzymatic activity on the reaction time (Fig. 2A, B), substrate concentration (Fig. 2C, D), and protein concentration (Fig. 2E, F). The formation of fluorescent products increased linearly up to 30 min for TMEM86A (Fig. 2A) and up to 60 min for TMEM86B (Fig. 2B). In order to avoid side reactions and to find a suitable condition for both isoforms detected together in tissues, we chose 30 min as the standard assay time. Regarding the substrate concentration, we performed the assays with increasing amounts (0–200 μM) of the two substrates. TMEM86A cleaved both the LPC[P] and LPE[P] substrate up to the highest concentration of 200 μM in a linear manner (Fig. 2C). For TMEM86B (Fig. 2D) we could detect a reproducible decline in the enzymatic activity at a LPC[P] substrate concentration of 200 μM. A similar finding, albeit for LPE[P], was published by Wu *et al.* in 2011 (4). The final substrate concentration in the assay mix was therefore set at 100 μM. When assaying with increasing protein amounts of the added homogenate, we started to reach a plateau phase at concentrations higher than 1 mg/ml (Fig. 2E for TMEM86A and Fig. 2F for TMEM86B) and therefore set our standard assay protein concentration to 1 mg/ml.

Lysoplasmalogenase activities were analyzed after three independent transfections of TMEM86A and TMEM86B to HEK293T cells on three independent days, and from these independent experiments, we could detect very reproducible values and therefore only minor day-to-day variabilities. Mock-transfected cells had activities of 2% or less of those found in cells transfected with wildtype TMEM86A or TMEM86B.

We also checked whether the Gly/Gly buffer at pH 7.2 used in a previously published lysoplasmalogenase assay coupled to NADH production by yeast alcohol dehydrogenase (4) was well suited for the reaction and compared it to Gly/Gly buffers with pH 7.6 or 8.0, as well as to potassium phosphate buffers at pH 6.4, 6.8, or 7.2. For both TMEM86 isoforms, Gly/Gly pH 7.2 yielded the highest activities confirming the data obtained by Wu *et al.* (4) (supplemental Fig. S2). To test the influence of substrate transacylation, we included the specific acyl-CoA–independent transacylase inhibitor SKF98625 in our initial assays, but unexpectedly, it inhibited the lysoplasmalogenase activity so strongly that we had to omit it from further studies (supplemental Fig. S3).

To exclude the possibility that lipids interfere with aldehyde production, we also performed a response curve to octadecanal in the presence and absence of lipid extract and found no differences in the amount of hydrazone formed (supplemental Fig. S4). Another question we addressed was whether PUFA oxidation occurred while lipid extracts were air-dried overnight. We compared this with drying in a stream of nitrogen gas, but, as shown in supplemental Fig. S5, no differences were detected.

### Differences in TMEM86A and TMEM86B substrate specificity

**Fig. 3:**
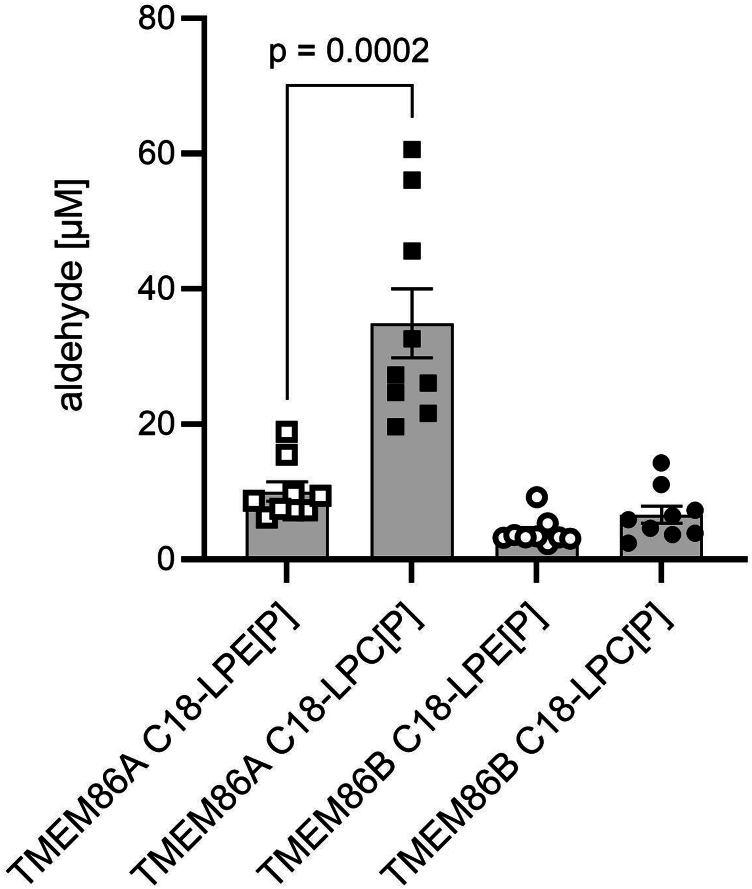
Dependence of human TMEM86A and TMEM86B-catalyzed aldehyde formation on ethanolamine (C18-LPE[P]) versus choline (C18-LPC[P]) head group in the lysoplasmalogen substrate. Data from all six panels from Figure 2 corresponding to the final assay standard conditions (30 min incubation in Fig. 2A, B, 100 μM as final substrate concentration in Fig. 2C, D and 1 mg/ml protein homogenate in Fig. 2E, F) were taken to generate this figure. The mean ± SEM for nine independent experiments is shown. Data were analyzed with two-tailed unpaired *t* test. LPC[P], lysoplasmenylcholine; LPE[P], lysoplasmenylethanolamine.

We checked for differences in substrate specificity of the two lysoplasmalogenases and plotted all activities obtained in Figure 2A–F under the final standard assay conditions of 100 μM of both substrates LPC[P] and LPE[P], 1 mg/ml of protein, and 30 min assay time (Fig. 3). While TMEM86B had no preference for one of the two substrates, TMEM86A metabolized the LPC[P] substrate 3.5-fold better than the ethanolamine analog (formed aldehyde from LPC[P]: 34.9 μM, from LPE[P]: 10.1 μM, *P* = 0.0002).

### Lysoplasmalogenase activities in mouse tissues

**Fig. 4:**
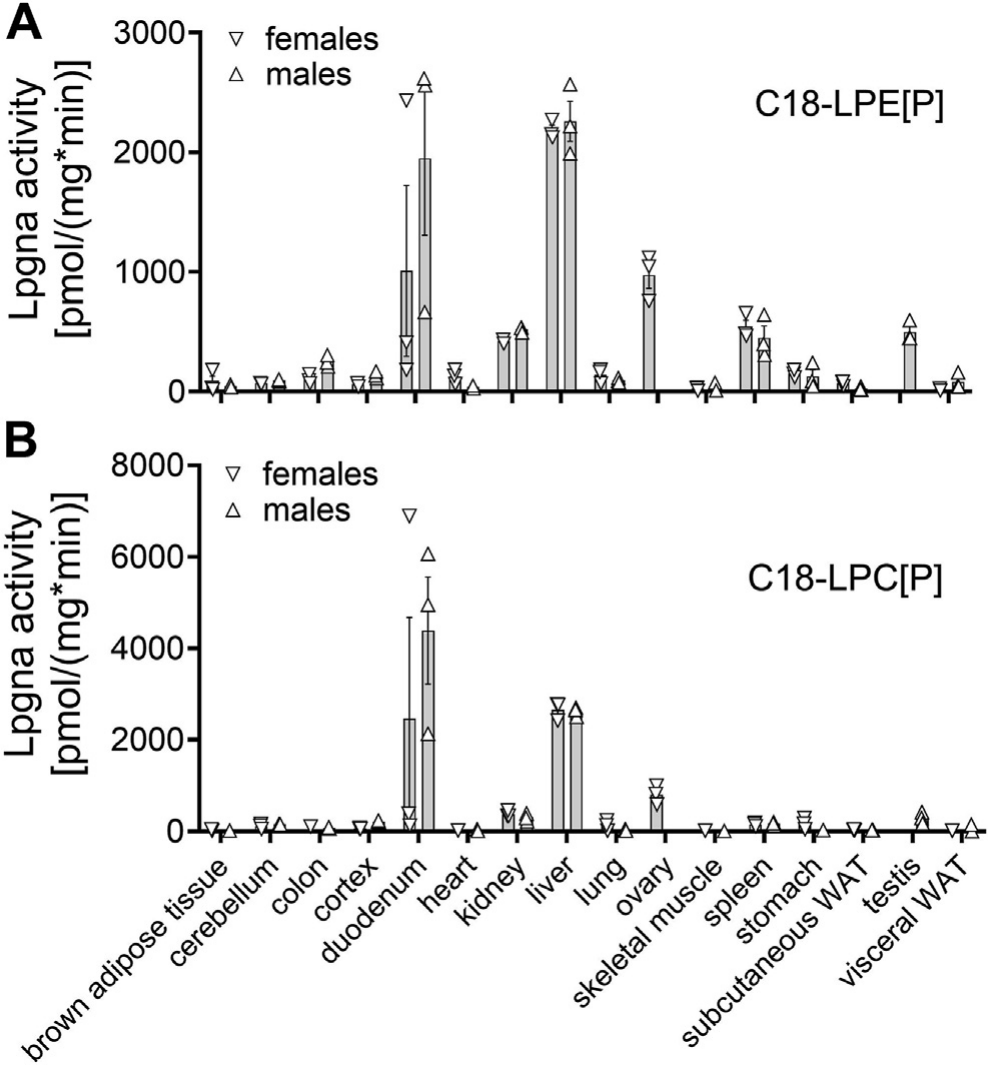
Lysoplasmalogenase activity distribution pattern in 16 female and male C57BL6/N wildtype mouse tissues. A: Tissue homogenates of three female (downward pointing triangles) and three male (upward pointing triangles) 12-week-old mice were homogenized and 1 mg/ml of protein subjected to the lysoplasmalogenase enzyme assay using (A) C18-LPE[P] or (B) C18-LPC[P]. The mean ± SEM for three independent experiments is shown. Sex differences were analyzed by two-way ANOVA followed by Tukey posthoc test, but no differences could be detected. LPC[P], lysoplasmenylcholine; LPE[P], lysoplasmenylethanolamine.

We tested whether our assay is useful in determining murine tissue lysoplasmalogenase activities. For this, we homogenized small tissue pieces of adult C57BL6/N wildtype mice (3 females and 3 males) with an Ultra Turrax and assayed 1 mg/ml protein homogenate under the same standard assay conditions as the transfected HEK293T cells. We found a comparable tissue distribution of lysoplasmalogenase activity for both the LPE[P] substrate (Fig. 4A) and LPC[P] substrate (Fig. 4B), with highest values obtained in liver and duodenum, followed by ovary, testes, spleen, and kidney. No significant differences in the activities between the two sexes were found. Many tissues, including the tested fat tissues, presented very low lysoplasmalogenase activity. As previous reports had shown TMEM86A to play a role in adipocyte metabolism, we enriched membranes from liver, sWAT, and vWAT and analyzed them again under the established standard assay conditions (supplemental Fig. S6). The activities detected in membrane fractions were twice as high as the activity in homogenates, but still several fold lower than those in the membranes obtained from liver (25-fold for LPE[P] and 15-fold for LPC[P]), indicating that lysoplasmalogenase activity is present in adipose tissue, but at very modest levels compared to liver, which has the highest activities.

### Analysis of eight proposed catalytically crucial residues in TMEM86A and TMEM86B

**Fig. 5:**
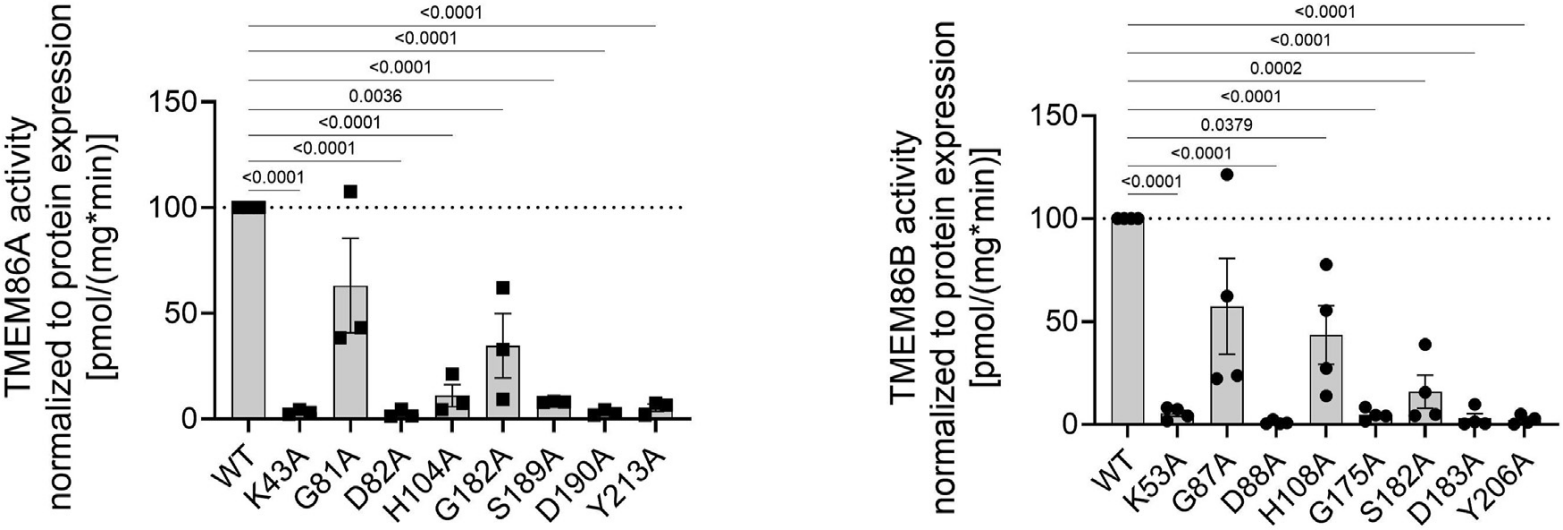
Analysis of eight point mutations in TMEM86A and TMEM86B. C-terminally tagged human TMEM86A (left panel) and human TMEM86B (right panel) expression plasmids, carrying the indicated mutations, were transiently transfected to HEK293T cells. Cells were harvested after 48 h and parallel wells analyzed in the lysoplasmalogenase enzyme assay, using C18-LPC[P]. Values were related to the amount of recombinant protein expressed (for representative Western blots see supplemental Fig. S7). Results show mean ± SEM for 3–4 independent experiments in percent relative to the respective wildtype. LPC[P], lysoplasmenylcholine.

Jurkovitz and coworkers proposed eight conserved and putative catalytically active amino acid residues in TMEM86A and TMEM86B based on sequence homology, that is, K43/53, G81/87, D82/88, H104/108, G182/175, S189/182, D190/183, and Y213/206 (first position refers to human TMEM86A and second refers to human TMEM86B) (8). We constructed these mutants by site-directed exchange to alanine in 6-myc–tagged wildtype constructs and related their activity (measured with the here presented enzymatic assay using C18-LPC[P]) to their protein expression, quantified by anti-myc Western blotting (Fig. 5, representative Western blots in supplemental Fig. S7). It emerged that, except for G81A in TMEM86A and G87A in TMEM86B, all of the predicted mutations affected enzymatic activity with most of them actually showing less than 10% of the wildtype activity.

Comparison of the protein expression of C-terminally 6x-myc–tagged wildtype TMEM86A and TMEM86B in transfected HEK293T cells by staining them with anti-myc antibody on the same membranes, revealed that both isoforms were expressed to the same extent (TMEM86A 101.1 ± 23.3% of TMEM86B, n = 3, representative Western blots in supplemental Fig. S7).

## Discussion

Degradation of the plasmalogen vinyl ether double bond has been postulated to follow two different pathways: either directly by the plasmalogenase cytochrome c, whose peroxidase activity is capable of hydrolytically cleaving the bond under oxidative conditions at the expense of molecular oxygen (1, 3), or after phospholipase A2-mediated deacylation to a lysoplasmalogen by the action of two lysoplasmalogenases, TMEM86A and TMEM86B (4, 5, 7). While the lysoplasmalogenase reaction of TMEM86B has been extensively studied using a purified protein (4), the enzymology of TMEM86A is less well characterized. Its classification as a lysoplasmalogenase is based on sequence homology (4), changes in total plasmalogen content (4) or lipid species concentration (5) upon manipulation of its expression, and UV absorption of NADH in transiently transfected HEK293T cells in a coupled enzymatic assay near the limit of detection (7).

We present here a novel assay for lysoplasmalogenase activity that utilizes reversed-phase HPLC–based quantification of the lipid aldehyde product after derivatization into a fluorescent dansylhydrazone. Previously developed optical assays used enzymatic oxidation of the aldehyde product of the enzymatic reaction and monitored the activity by optical absorption of the thus formed NADH (4, 7). Compared to these optical assays, our method provides much higher sensitivity, due to the intense fluorescence of the dansylhydrazone formed by derivatization of the aldehyde yielded from the lysoplasmalogenase reaction, which can be sensitively quantified in small volumes by reversed-phase HPLC and fluorescence detection.

Using this assay, we were able to clearly confirm lysoplasmalogenase activity of TMEM86A in transiently transfected HEK293T cells. In this system, TMEM86A showed a 3.5-fold higher turnover of C18-LPC[P] than C18-LPE[P] under the standard conditions of our assay. In contrast, TMEM86B degraded C18-LPE[P] and C18-LPC[P] at similar rates (Fig. 3), which was also observed with the purified enzyme and the optical absorption assay (4). Another striking difference between TMEM86A and TMEM86B was the profound inactivation of TMEM86B by 200 μM C18-LPC[P] (Fig. 2D), which was not observed for TMEM86A (Fig. 2C). A possible interpretation of this is that TMEM86B may be more sensitive to the detergent effect of C18-LPC[P] than TMEM86A, for which this compound is the preferred substrate.

As a first insight into the catalytic activity of both TMEM86A and TMEM86B, eight potentially critical residues were postulated based on homology (8). We mutated these positions individually to alanine in both proteins and analyzed the effect on activity and protein expression (Fig. 5, supplemental Fig. S7). Our analysis clearly showed that these residues were indeed crucial for the activity, with the exception of glycine-81 in TMEM86A (corresponding to glycine-87 in TMEM86B). Except for histidine-104 and glycine-182 (in TMEM86A) and histidine-108 and serine-182 (in TMEM86B) all mutations decreased the activity to less than 10% of wildtype. Cho *et al.* had already tested the two aspartate residues at positions 82 and 190 in TMEM86A and had also found them to be absolutely necessary for the catalysis (5). Further studies will be needed to understand the structure-function implications of these residues. To assess the effect of the mutations on protein expression, we utilized 6x myc–tagged proteins as in our previous studies (12, 13, 14). This strategy enabled a direct comparison of the expression of both wildtype isoforms using an anti-myc antibody, revealing that wildtype TMEM86A and TMEM86B exhibited equal protein expression levels (supplemental Fig. S7).

In murine tissues (Fig. 4), we quantified the highest activities in the duodenum and liver, consistent with a possible role in the processing of dietary plasmalogens, followed by the kidney and spleen; the primary reproductive organs ovary and testis also showed distinctly high activity. Interpretation of these results is difficult given our limited knowledge of the role of lysoplasmalogens in metabolism, other than as intermediates in plasmalogen degradation. We know from previous studies that the liver contains only small amounts of plasmalogens and lysoplasmalogens (10, 15, 16). Based on our data on high lysoplasmalogenase activity in the liver, we conclude that the liver is not the main organ responsible for supplying other parts of the body with these lipids, but rather that these are most likely synthesized locally, depending on the amount required in individual tissues (16).

Lysoplasmalogenase activity was low in most other organs, including adipose tissue although TMEM86A has been described to play a role in murine adipocytes (5). We prepared membrane-enriched fractions of sWAT, vWAT, and liver and were able to detect higher levels of lysoplasmalogen-degrading activity than in homogenates, but these were very low compared to liver. However, this is in good agreement with the paper mentioned above, as the authors reported strongly induced TMEM86A expression only in mice fed a high-fat diet. In contrast, mice on a control diet - like those in our study - exhibited only low TMEM86A expression levels (5).

The proposed involvement of TMEM86A in severe acute respiratory syndrome coronavirus 2 resistance (6), protein kinase A–dependent energy metabolism (5), sterol-dependent membrane remodeling in macrophages (7), and keratinocyte differentiation (17) suggests that we may learn new roles for lysoplasmalogens and their degradation by TMEM86A and TMEM86B. This is extremely urgent given the association of reduced levels of plasmalogens with neurodegenerative diseases such as Alzheimer's and Parkinson's disease, and the existence of severe inherited disorders in children suffering from total ether lipid deficiency. A more detailed understanding of the regulation of plasmalogen degradation and the enzymes involved may provide a successful strategy to modulate their levels and thereby benefit patients. We are convinced that our novel sensitive assay will help to advance this area of research.

## Data availability

All data described are contained in the article.

## Supplemental data

This article contains supplemental data.

## Conflicts of interest

The authors declare that they have no conflicts of interest with the contents of this article.

## Supplementary data

Kummer_et_al_supplements

CRediT author statement
