# Supplementary material for: Functional characterization of TMEM86A and TMEM86B mutants by a novel lysoplasmalogenase assay
Source: J Lipid Res. 2025 Feb 28;66(4):100766. doi: 10.1016/j.jlr.2025.100766 (PMC11994398; doi:10.1016/j.jlr.2025.100766)
Supplement: Kummer_et_al_supplements [file mmc1.docx]

**Supplementary figures**


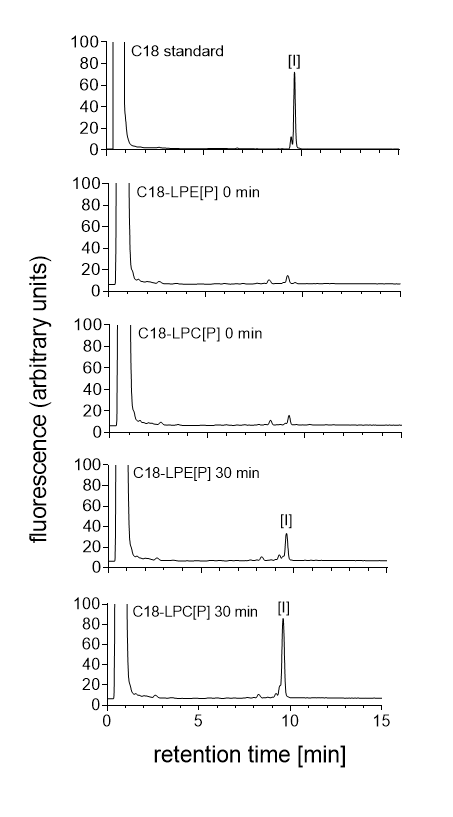


*Supplementary Fig. S1: Representative chromatograms of lysoplasmalogenase assays using octadecanal as external standard. First panel, octadecanal was derivatized with dansylhydrazine and the corresponding C18-hydrazone eluted from the reversed-phase HPLC as peak [I]. Second and third panel, TMEM86A-transfected HEK293T cell homogenates were incubated with 100 µM C18-LPE[P] or C18-LPC[P] and the reaction was immediately stopped (0 min). Panel four and five show typical chromatograms of the same samples shown in the second and third panel after 30 min incubation with C18-LPE[P] or C18-LPC[P] yielding formation of the C18-hydrazone [I].*

**

*Supplementary Fig. S2: Dependence of lysoplasmalogen cleavage by human TMEM86A (squares) and TMEM86B (circles) on buffer composition and pH using 100 µM LPE[P] (open symbols, left panel) or LPC[P] (filled symbols, right panel) as substrates. The following buffers were used for the lysoplasmalogenase assay: Gly/Gly pH 7.2, 7.6 and 8.0, and potassium phosphate (KPi) pH 6.4, 6.8 and 7.2. Protein concentration was 1 mg/ml, all assays were stopped after 30 min, lipids extracted and aldehydes derivatized with dansylhydrazine to the respective fluorescent hydrazones. The amount of aldehyde was calculated from the area of the peak at the correct retention time and related to the aldehyde formed in Gly/Gly pH 7.2. The mean ± SEM for three independent experiments is shown.*

*Supplementary Fig. S3:* *Dependence of lysoplasmalogenase activity in wildtype mouse liver on the specific acyl-CoA-independent transacylase inhibitor SKF98625 added in a final concentration of 10, 30 and 100 nM in assays using 100 µM LPE[P] (open circles) or LPC[P] (filled circles) as substrates (1 mg/ml protein concentration, 30 min incubation). Lipids were extracted and aldehydes derivatized with dansylhydrazine to the respective fluorescent hydrazone. The amount of aldehyde was calculated from the area of the peak at the correct retention time. The mean ± SEM for three independent experiments is shown.*

*Supplementary Fig. S4: Response curve of octadecanal derivatization on lipids present in lipid extract. 0, 0.3, 1 and 3 µM octadecanal were derivatized in absence (down-pointing filled triangles) and presence (up-pointing open triangles) of a lipid extract prepared from HEK293T cells, analyzed by reversed-phase HPLC and the amount of aldehyde calculated from the area of the peak at the correct retention time. The mean ± SEM for three independent experiments is shown.*

*Supplementary Fig. S5: Comparison of overnight air-drying of lipid extracts with drying under a constant steam of gaseous nitrogen. HEK293T cells transfected with human TMEM86A (squares) or TMEM86B (circles) were assayed with 100 µM LPE[P] (left panel, open symbols) or LPC[P] (right panel, filled symbols) as substrates and 1 mg/ml protein concentration. After 30 min incubation, the reaction was stopped and lipids were extracted. Organic solvent of the extracts was left overnight to air-dry (O/N) or was dried under a stream of gaseous nitrogen (N_2_). Aldehydes were then derivatized with dansylhydrazine to the respective fluorescent hydrazones and the amount of aldehyde calculated from the area of the peak at the correct retention time. The mean ± SEM for three independent experiments is shown.*

*Supplementary Fig. S6: Lysoplasmalogenase activity in membranes prepared from liver and two adipose tissues, vWAT and sWAT, of C57BL6/N wildtype mice. Tissues were harvested from three 12-week-old male wildtype mice and membranes were prepared by differential centrifugation. 0.15-0.5 mg/ml of protein was subjected to the lysoplasmalogenase enzyme assay using 100 µM C18-LPE[P] (open symbols) or C18-LPC[P] (filled symbols). The mean ± SEM for three independent experiments is shown.*


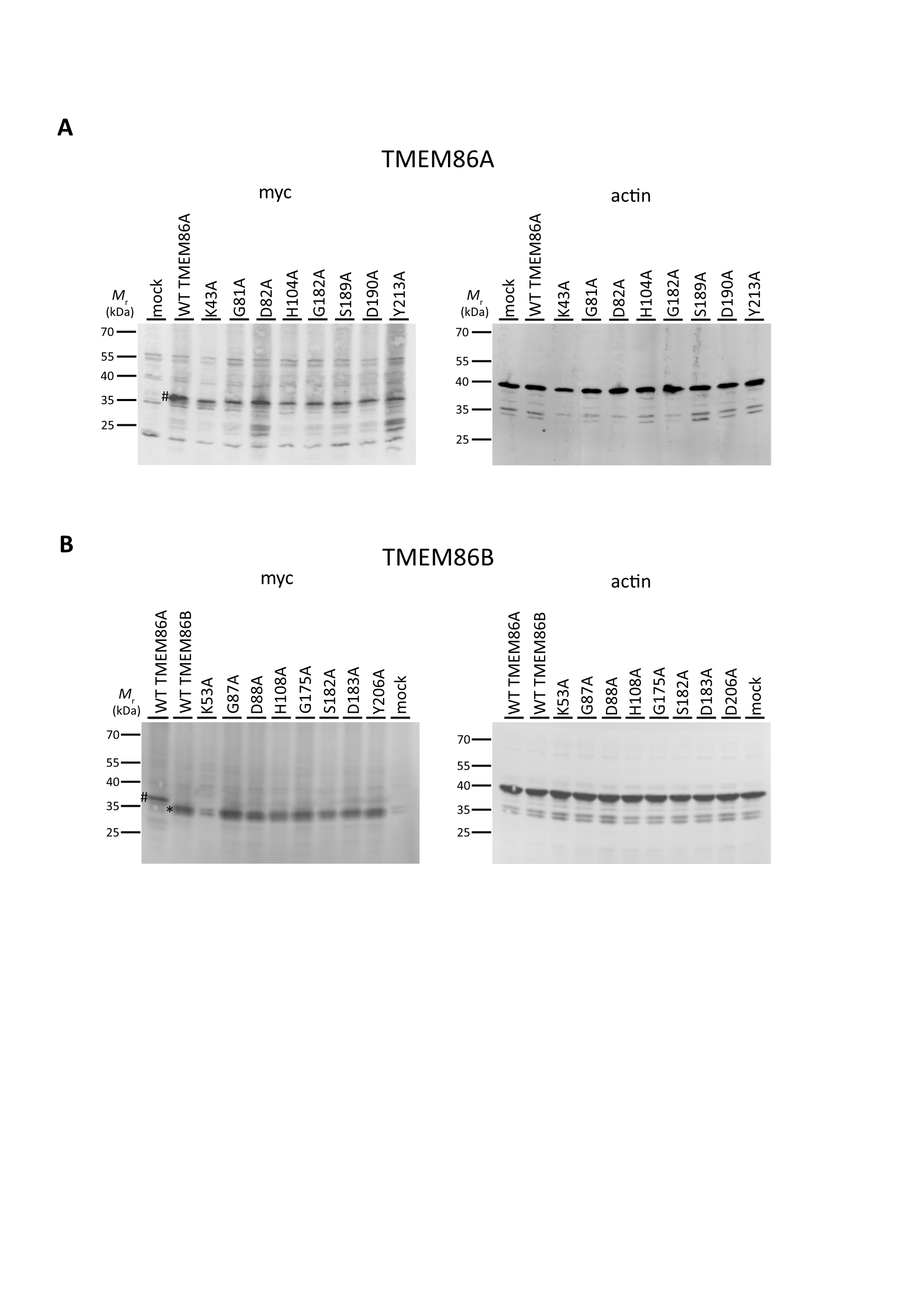


*Supplementary Fig. S7: Representative Western blots of TMEM86A (A) or TMEM86B (B) point mutations after transfection of HEK 283T cells. Left panels: anti-myc stained blots. Right panel: anti-actin stained blots. Mock indicates cells transfected with GFP as transfection control. Hash denotes the band of myc-tagged TMEM86A (calculated molecular mass: 39 kDa) and star the band of myc-tagged TMEM86B (calculated molecular mass: 37 kDa). The same apparent molecular masses apply to the distinct mutations. One representative Western blot of 3-4 is shown.*

*Supplementary Table 1: Forward and reverse primer sequences for introduction of mutations in human TMEM86A and human TMEM86B by QuikChange® site-directed mutagenesis.*

| ***Human TMEM86A*** | ***Forward primer (5’ 🡪 3’)*** | ***Reverse primer (5’ 🡪 3’)*** |
| --- | --- | --- |
| *K43A* | GGTCAGCACCCTCATCGCATGCCTGCCTATCTTCTG | CAGAAGATAGGCAGGCATGCGATGAGGGTGCTGACC |
| *G81A* | CTTGTCTTCTCTGCAGTAGCTGACGCCTTCCTCATC | GATGAGGAAGGCGTCAGCTACTGCAGAGAAGACAAG |
| *D82A* | GTCTTCTCTGCTGTAGGCGCCGCATTCCTCATCTGG | CCAGATGAGGAATGCGGCGCCTACAGCAGAGAAGAC |
| *H104A* | CTGATGTTTGCTGTGACCGCCATGTTCTACGC | GCGTAGAACATGGCGGTCACAGCAAACATCAG |
| *G182A* | GCTGGCAGTGCTGCACTCTTCTTTATCATCTCAG | CTGAGATGATAAAGAAGAGTGCAGCACTGCCAGC |
| *S189A* | CTCTTCTTTATCATCGCAGACCTGACCATCGCCCTCAAC | GTTGAGGGCGATGGTCAGGTCTGCGATGATAAAGAAGAG |
| *D190A* | GCACTCTTCTTTATCATCTCAGCGTTAACCATCGCCCTCAACAAATTCTG | CAGAATTTGTTGAGGGCGATGGTTAACGCTGAGATGATAAAGAAGAGTGC |
| *Y213A* | GCTTATCATGTCCACCGCCTACGTAGCCCAGATGCTCGTC | GACGAGCATCTGGGCTACGTAGGCGGTGGACATGATAAGC |
| ***Human TMEM86B*** |  |  |
| *K53A* | GCTGCCCTGGTCGCATGCCTGCCCGTCCTC | GAGGACGGGCAGGCATGCGACCAGGGCAGC |
| *G87A* | CCCTTGTGTGCTCTGCAGTGGCGGACGCTTGCC | GGCAAGCGTCCGCCACTGCAGAGCACACAAGGG |
| *D88A* | CTCGGCTGTGGGCGCCGCTTGCCTCATC | GATGAGGCAAGCGGCGCCCACAGCCGAG |
| *H108A* | CGCCTTTGCCACGGCCGCCCTCCTCTACGTC | GACGTAGAGGAGGGCGGCCGTGGCAAAGGCG |
| *G175A* | GGCTGGGCCGCGCTGCTCTTCACG | CGTGAAGAGCAGCGCGGCCCAGCC |
| *S182A* | CGCTGCTCTTCACGCTAGCTGATGGAGTGCTGGCCTGGGACACC | GGTGTCCCAGGCCAGCACTCCATCAGCTAGCGTGAAGAGCAGCG |
| *D183A* | CTCTTCACGCTCTCTGCCGGCGTGCTGC | GCAGCACGCCGGCAGAGAGCGTGAAGAG |
| *Y206A* | GACCACCGCATATGCTGCCCAGCTCCTCATCACACTG | CAGTGTGATGAGGAGCTGGGCAGCATATGCGGTGGTC |
