# Supplementary material for: Functional characterization of TMEM86A and TMEM86B mutants by a novel lysoplasmalogenase assay
Source: J Lipid Res. 2025 Feb 28;66(4):100766. doi: 10.1016/j.jlr.2025.100766 (PMC11994398; doi:10.1016/j.jlr.2025.100766)
Supplement: CRediT author statement [file mmc2.docx]

***CRediT author statement***

**Denise Kummer:** Investigation, Resources, Writing - Reviewing and Editing. **Ilaria Dorigatti:** Investigation, Resources, Writing - Reviewing and Editing. **Theresia Dunzendorfer-Matt:** Formal analysis, Writing – Reviewing and Editing. **Georg Golderer:** Conceptualization, Formal analysis, Writing - Reviewing and Editing, Supervision. **Ernst R. Werner:** Conceptualization, Methodology, Formal analysis, Writing - Original Draft, Writing - Reviewing and Editing, Visualization, Supervision, Project administration. **Katrin Watschinger:** Conceptualization, Formal analysis, Writing - Original Draft, Writing - Reviewing and Editing, Visualization, Supervision, Project administration, Funding acquisition.

|  |  |
| --- | --- |
|  |  |
|  |  |
|  |  |
|  |  |
|  |  |
|  |  |
|  |  |
|  |  |
|  |  |
|  |  |
|  |  |
|  |  |
|  |  |
